# SEVERE ACUTE TOXIC EXPOSURES IN CHILDREN AND ADOLESCENTS: CASE SERIES

**DOI:** 10.1590/1984-0462/2021/39/2019262

**Published:** 2020-07-03

**Authors:** Daniela Brianne Martins dos Anjos, Adriana Safioti Toledo Ricardi, Carla Fernanda Borrasca Fernandes, Camila Carbone Prado, Eduardo Mello De Capitani, Fábio Bucaretchi

**Affiliations:** a Universidade Estadual de Campinas, Campinas, SP, Brazil.

**Keywords:** Poison Control Center, Poisoning, Envenomation, Pediatrics, Centro de Controle de Intoxicações, Envenena­mento, Pediatria

## Abstract

**Objective:**

To describe a case series of severe acute toxic exposures (SATE) in individuals <20 years old followed-up by a regional Poison Control Center (PCC).

**Methods:**

Descriptive cross-sectional study. All patients who were <20yo and classified as score 3 (severe) and 4 (fatal) following Poisoning Severity Score were included for analysis. According to the outcome, patients were classified as PSS 3 when they developed intense clinical manifestations with risk of death or important sequelae; and as PSS 4 when death had resulted from direct cause or complication of the initial exposure. The data of patients were obtained from the Brazilian electronic database system (DATATOX).

**Results:**

During the biennium 2014-2015, Campinas PCC followed up 5,095 patients <20yo, with 30 being classified as SATE (PSS=3, n=24; PSS=4, n=6). The exposures circumstances were unintentional (15); intentional (14; suicide attempt = 11; street drugs consumption = 3); and not explained (1). The exposures were significantly more frequent in adolescents >14yo (n=17; p<0.01). The involved agents were venomous animals (8; scorpions=5); medicines (8; miscellaneous=6); chemicals (6); illegal rodenticides containing acetylcholinesterase inhibitors (*chumbinho*, 4); drugs of abuse (3); button battery (1). Three patients evolved with sequels (esophageal stricture post-corrosive ingestion). The median length of hospital stay was 6 days (IQR: 5-12 days); 26 patients were treated in intensive care units, and 22 of them needed mechanical ventilation; 12, inotropic/vasopressors; and 3, renal replacement therapy.

**Conclusions:**

Scorpion stings and poisonings caused by medicines and chemicals were the main causes of SATE. The SATE were significantly more frequent in adolescents, due to deliberate self-poisoning.

## RESUMO

**Objetivo:**

Descrever uma série de casos de exposições tóxicas agudas graves (ETAG) em pacientes <20 anos seguidos por um Centro de Informação e Assistência Toxicológica (CIATox) regional.

**Métodos:**

Estudo descritivo de corte transversal. Incluídos no estudo todos os casos classificados como escore 3 (graves) e 4 (fatais) de acordo com o escore de gravidade de intoxicações (*poisoning severity score -* PSS). Segundo o PSS, os casos são classificados em relação ao desfecho como *escore 3* quando os pacientes desenvolveram manifestações clínicas intensas, com risco de morte ou que resultaram em sequelas importantes; e *escore 4* quando a morte foi resultado de causa direta ou por complicação da exposição. Os dados analisados foram obtidos da base eletrônica brasileira do Sistema Nacional de Informações Tóxico-Farmacológicas DATATOX.

**Resultados:**

No biênio 2014-2015 o CIATox de Campinas atendeu 5.095 casos de pacientes <20 anos, dos quais 30 foram classificados como ETAG (PSS=3, n=24; PSS=4, n=6). Quanto à circunstância, 15 foram acidentais, 14 intencionais (11 por tentativas de suicídio e três por abuso de drogas) e um de causa não esclarecida, sendo significativamente mais frequentes nos adolescentes >14 anos (n=17; p<0,01). Os grupos de agentes envolvidos foram: animais peçonhentos (8; escorpiões=5); medicamentos (8; associações=6); produtos químicos de uso domiciliar/industrial (6); rodenticidas inibidores da colinesterase de uso ilegal (chumbinho=4); drogas de abuso (3); e bateria no formato de disco (1). Três pacientes evoluíram com sequelas (estenose esofágica pós-ingestão de corrosivos). O tempo mediano de internação foi de seis dias (mediana, quartis e intervalo interquartil=5-12 dias), sendo 26 pacientes admitidos em unidades de cuidados intensivos, dos quais 22 necessitaram de ventilação mecânica, 12 de inotrópicos/vasopressores e três de terapia de substituição renal.

**Conclusões:**

Escorpionismo e intoxicações por medicamentos e por produtos químicos de uso domiciliar/industrial foram as principais causas de ETAG, sendo elas significativamente mais frequentes em adolescentes, principalmente por tentativas de suicídio.

## INTRODUCTION

Poison Control Centers (PCC) are public reference services in clinical toxicology, of regional or national scope, with assistance on permanent duty by telephone and/or in person,^1^ with health professionals being its main requesters in Brazil.

The services offered by PCCs involve human and animal exposures, including information on any type of intoxication/poisoning. In general, the service aims to assist the health professional in the clinical management of the intoxicated patient, supported by information contained in electronic databases of international toxicological information constantly updated and in evidence available in the specialized literature, helping to prevent/reduce damage and lower health costs in this activity area.^1^ ^,^ ^2^ ^,^ ^3^ ^,^ ^4^ ^,^ ^5^ ^,^ ^6^ ^,^ ^7^

Among the cases registered in PCCs, the toxic exposures in pediatrics stand out due to their frequency.^6^ ^,^ ^7^ Despite this consideration, studies with the sole objective of evaluating case series of cases of severe acute toxic exposures (SATE) in children and adolescents, including epidemiological aspects and clinical management, have not been previously described in Brazil. From the above, this work aimed to analyze a case series SATE in children and adolescents followed by a PCC of regional reference for a population of six million inhabitants in the Southeastern region of Brazil, including a summary description of all fatal cases notified.

## METHOD

A descriptive cross-sectional study of pediatric care (0 to 20 years old) of SATE was carried out, followed by the Campinas PCC (exclusive and face-to-face phone calls), from January 1^st^, 2014 to December 31^st^, 2015. The data for analysis were obtained from the National System for Toxic-Pharmacological Information (Datatox’s electronic base), from the Brazilian Association of Toxicological Information and Assistance Centers (*Associação Brasileira de Centros de Informação e Assistência Toxicológica* - ABRACIT). The notification of cases in the Datatox system is not mandatory and is carried out only by those PCCs associated to ABRACIT.

Only cases classified as severe and fatal to the outcome were included in the study group according to the poisoning severity score (PSS) guidelines, which are similar to the one used to close the case in the Datatox system. According to the PSS, cases are classified according to their evolution, such as:

- Asymptomatic (PSS=0).
- Mild (PSS=1): discrete and transient clinical manifestations that are quickly solved.
- Moderate (PSS=2): more pronounced, more prolonged or more systemic clinical manifestations that usually require treatment.
- Severe (PSS=3): intense clinical manifestations, with risk of death or resulting in important sequelae.
- Fatal (PSS=4): as a direct cause or complication of exposure.^8^

Demographic and clinical variables were analyzed, including age range, intentionality, agents involved, treatments used and evolution. The non-parametric statistical analysis of some variables was performed, such as position measurements (median, quartiles and interquartile range - IQR), and, for the statistical analysis of the difference in the distribution of values between independent and related samples, the chi-squared test was applied, adopting p<0.05 as significant. All toxicological analyzes mentioned in the results took place at the Campinas PCC Analytical Toxicology Laboratory.

The present study is part of a broader project inserted in *Plataforma Brasil* and approved by the Research Ethics Committee (CEP) of Universidade Estadual de Campinas (Unicamp) - Certificate of Presentation for Ethical Appreciation (CAAE) No. 43725215.9.0000.5404.

## RESULTS

During the study period (2014-2015), the Campinas PCC served 5,095 individuals with toxic exposures, aged <20, mainly in the age group between 1-4 years old (64.2%; Table 1). In the rescue of cases classified as severe and fatal in the Datatox system, 41 electronic medical records with this classification were detected. These records were reviewed by four authors (DBMA, ASTR, CCP and FB), in a non-blind manner, who, by consensus, concluded that 30 patients met the criteria for the outcome classification by the PSS as score 3 (n=24) and score 4 (n=6) (Table 1).

**Table 1:** Final classification of toxic exposure cases followed by the Campinas Poison Control Center (PCC) (2014 and 2015), in children and adolescents, according to the outcome and age group (years old).

Outcome (PSS)/age group (years old)	<1	1-4	5-9	10-14	15-19	Total	%
Asymptomatic (PSS=0)	250	2,027	346	212	273	3,108	61.0
Mild (PSS=1)	86	675	241	208	301	1,511	29.7
Moderate (PSS=2)	22	76	40	37	46	221	4.3
Severe (PSS=3)	1	4	2	2	15	24	0.5
Fatal with causal nexus (PSS=4)	0	2	2	0	2	6	0.1
Potentially toxic exposures without monitoring	3	10	1	1	1	16	0.3
Death from another cause	3	2	1	1	0	7	0.1
Ignored	14	98	26	27	37	202	4.0
Total (%)	379 (7.4)	2,894 (56.8)	659 (12.9)	488 (9.6)	675 (13.2)	5,095 (100)	100

PSS: poisoning severity score.

According to the data in Table 1, when comparing the proportions between the groups of severe/fatal cases (PSS=3 and 4) and non-severe (PSS=0, 1 and 2) in relation to the different age subgroups, it was possible to find that SATE were significantly more frequent in adolescents >14 years old (<1 year old, 1/358; 14 years old, 6/2,778; 5-9 years old, 4/627; 10-14 years old, 2/457; 15-19 years old, 17/620; p<0.001). As to the demographic characteristics of the study group (n=30), most were male (n=22), with a median age of 15 years (IQR=7-17 years), and that most of the consultations happened through exclusive telephone follow-up (n=26). As for the circumstance of exposure, in 15 cases it was classified as accidental, and in 14 cases, as intentional, the latter occurring only in adolescents between 15 and 19 years old (suicide attempts, n=11; psychoactive substance abuse, n=3) (Table 2). In the fatal case of a 9-year-old girl, the cause of death was not clarified, with the possibility of mistreatment/neglect or suicide considered (see synopsis of case 4). The median hospital stay was six days (IQR=5-12 days), and 26 patients were admitted to intensive care units.

**Table 2:** Cases of severe acute toxic exposures in children and adolescents according to age group (years old) in relation to the circumstance of exposure, group of agents and evolution.

Age group (years old)	<1	1-4	5-9	10-14	15-19	Total
Circumstance of exposure						
Accidental	1	6	3	2	3	15
Intentional	0	0	0	0	14	14
Not clarified	0	0	1	0	0	1
Group of agents						
Venomous animals	0	0	3	2	3	8
Medications	1	0	1	0	6	8
Household or industrial chemicals	0	5	0	0	1	6
Rodenticides for illegal use (*chumbinho*)	0	0	0	0	4	4
Drugs of abuse	0	0	0	0	3	3
Button battery	0	1	0	0	0	1
Evolution						
Cure	1	2	2	2	14	21
Anatomical sequelae (caustic esophageal stenosis)	0	2	0	0	1	3
Fatal	0	2	2	0	2	6

Table 2 shows the groups of agents involved according to the different age subgroups, and Table 3, the specific agents. Such data indicate that venomous animals, such as scorpions and rattlesnakes, medicines (especially anticonvulsants and antidepressants), household/industrial chemicals, cholinesterase-inhibiting rodenticides (*chumbinho*) and recreational drugs of illegal use were the main agents involved. The common name in Portuguese, *chumbinho*, (“small lead pellets”) derives from the physical appearance of the product, which generally consists of small, dark grey, regular-shaped granules. Table 4 shows the main clinical findings, with emphasis on neurological depression, hypotension/shock and respiratory failure, with 73% of patients requiring mechanical ventilation and 40%, vasopressors/inotropes.

**Table 3:** Cases of severe acute toxic exposures in children and adolescents according to the agents involved and the evolution (sequelae and deaths).

Agents involved, including associations	N	Sequelae	Deaths
Venomous animals			
Scorpions			
Unidentified species (possibly *Tityus serrulatus*)	4	0	0
*Tityus serrulatus*	1	0	1
Unidentified rattlesnake (possibly *Crotalus durissus terrificus*)	3	0	0
Medications			
Brimonidine	1	0	0
Carbamazepine	1	0	0
Carbamazepine and diazepam	1	0	0
Captopril and sertraline	1	0	0
Amitriptyline, diazepam and paracetamol	1	0	0
Valproic acid, haloperidol and promethazine	1	0	1
Chlordiazepoxide, lamotrigine, sertraline, venlafaxine and desogestrel	1	0	0
Carbamazepine, lithium carbonate, chlorpromazine, diazepam, phenytoin and phenobarbital	1	0	0
Household or industrial chemicals			
Corrosive			
Sodium hydroxide	2	2	0
Sodium dodecylbenzenesulfonate	1	0	1
Chlorine for swimming pool	1	1	0
Kerosene	1	0	0
Hydrocarbon solvent (derivatives of N-hexane)*	1	0	1
Rodenticides of clandestine use (chumbinho)			
Cholinesterase inhibitors **	4	0	1
Drugs of abuse			
Cocaine and tetrahydrocannabinol	1	0	1
Phenylethylamine (NBOMe)	1	0	0
Inhalants†	1	0	0
Button battery	1	0	0
Total	30	3	6

*The product accidentally ingested/inhaled was a pyrethroid insecticide, but the death resulted from exposure to the solvent present in the product (see case 1 synopsis); **not investigated by laboratory analysis if the cholinesterase inhibitor was a carbamate or an organophosphate; † presence of benzene and cyclohexane confirmed in the blood sample and chloroform in the urine sample.

**Table 4:** Main clinical findings observed during admission and evolution of the 30 cases of severe acute toxic exposures, including syndromic diagnoses and treatments employed.

Clinical, laboratory findings and treatments employed	n
Neuromuscular changes	
Neurological depression	13
Cholinergic syndrome	4
Myasthenic syndrome	3
Seizures	3
Severe rhabdomyolysis (total CK>10,000 U/L)	3
Psychomotor agitation	2
Serotonin syndrome	1
Cardiovascular changes	
Hypotension/shock	12
Myocardial injury (increase in serum CKMB and troponin)	6
Corrected QT interval widening (>440 ms)	4
Changes in the ST segment (depression and/or elevation)	4
Myocardial dysfunction (LV ejection fraction <56%)	3
Respiratory changes	
Respiratory failure	11
Pneumonia	8
Acute pulmonary edema	4
Others	
Acute kidney injury	4
Severe esophageal injury	4
Coagulopathy	2
Treatments used	
Parenteral hydration	22
Mechanical ventilation	22
Vasopressors / inotropes	12
Continuous atropine infusion	4
Anti-scorpionic antivenom	4
Anticrotalic antivenom	3
Renal replacement therapy	3
Multiple doses of activated charcoal	3
Prolonged parenteral nutrition	2
Intravenous N-acetylcysteine	1

CK: creatine kinase; LV: left ventricle.

Among the three cases that evolved with sequelae (esophageal stricture after ingestion of corrosives), two were accidental (sodium hydroxide) and one was due to a suicide attempt (chlorine-based sanitizing for use in swimming pools). In one of the accidental cases with sodium hydroxide (preparation of homemade soap; one-year-old boy), the patient showed signs of acute respiratory failure and was submitted to tracheostomy at the local service, complicated by injury to the anterior esophageal wall, evolving, besides caustic stricture, with tracheoesophageal fistula, being hospitalized for 28 days. In addition, a one-year-old boy presented a circumferential lesion of the esophagus after ingesting a button battery, evolving with transient esophageal stenosis reversed after two endoscopic dilations.

Considering the estimated population for the administrative region of Campinas in the years 2014 and 2015,^9^ a lethality rate for toxic events of 0.24 and 0.17 cases/100 thousand inhabitants was obtained for the age groups <5 and <20 years old, respectively.

Here is a synopsis of the six fatal cases:

- Case 1 (telephone follow-up): boy, one year old, admitted 90 minutes after accidental ingestion / inhalation of pyrethroid insecticide, containing hydrocarbon solvents in its formulation. He evolved with seizures, a reversed cardiorespiratory arrest and respiratory failure due to pulmonary edema (chemical pneumonitis), dying 24 hours after exposure. Death was attributed to exposure to hydrocarbons present in the product, with identification of N-hexane derivatives in blood samples collected before death (gas chromatography-mass spectrometry - GC-MS).
- Case 2 (telephone follow-up): boy, two years old, admitted two hours after accidental ingestion of a sip of an alkaline degreaser (sodium dodecylbenzenesulfonate, pH=12.5-13.5), which was stored in a glass of alcohol. The boy showed drooling and signs of respiratory difficulty. In the service of origin, gastric lavage and administration of activated charcoal and atropine were performed. The PCC was then contacted, warning about the formal contraindications of the procedures that were performed, considering the ingestion of a corrosive. The patient died about four hours after exposure. Necropsy findings revealed signs of chemical burn in the entire esophageal mucosa and in the stomach, with the presence of activated charcoal inside the stomach, without signs of esophageal or gastric perforation, in addition to hyperemia of the tracheal mucosa, presence of activated charcoal in the trachea and lungs with an inflammatory aspect, with the discharge of a large amount of secretion from the tracheobronchial tree, suggesting aspiration pneumonia as a possible cause of death.
- Case 3 (face-to-face): girl, nine years old, admitted to the emergency care unit about 40 minutes after a *Tityus serrulatus* scorpion sting in her left hand, with intense local pain, psychomotor agitation and visual blurring, treated with local anesthetic infiltration and analgesics intravenously. Despite the improvement in pain, she maintained agitation, progressing with hyperemesis, tachypnea, tachycardia, arterial hypertension, diaphoresis and hypothermia, and was treated with antiscorpionic antivenom (2 hours post-sting). She evolved with pulmonary edema and refractory cardiogenic shock, and died on the second day of hospitalization (D2);
- Case 4 (telephone follow-up): girl, nine years old, admitted with neurological depression, signs of shock, nasal bleeding, bradycardia, hypothermia (33°C), hypoglycemia (28 mg/dL), acute kidney injury and QT interval prolongation on the electrocardiogram. According to information provided by her grandmother, the child possibly ingested, the night before, haloperidol, clonazepam and promethazine, grandmother’s medications, who found the empty cartons on the floor. The grandmother reported that, since then, the child has been sleeping, and she took the girl to the hospital only the next morning, because she would not wake up. During evolution, she response partially to the bolus glucose injection, maintaining signs of shock and hypothermia. Despite supportive measures, the patient died six days after admission (signs of brain death on the fourth day). Toxicological screening on GC-MS was positive for haloperidol, promethazine and valproic acid and negative for glibenclamide, metformin and benzodiazepines. It was not possible to search for other oral hypoglycemic agents in addition to glibenclamide in the sample sent (urine), due to the lack of standards in the laboratory for this analysis.
- Case 5 (telephone follow-up): boy, 15 years old, recent cocaine user, found unconscious on the street. During pre-hospital care, he had a seizure, was sedated and intubated. Upon admission, he had a mild tachycardia, hypertension and nosebleed, and mouth bleeding. Admission tests showed positive toxicological screening for cocaine and tetrahydrocannabinol and a slight increase in total creatine kinase (CK). He developed arterial hypertension, severe acute kidney injury (hemodialysis on the fourth day), pneumonia and hemorrhagic stroke (on the 13^th^ day), and died on the 16^th^ day.
- Case 6 (telephone follow-up): boy, 17 years old, admitted with neurological depression, severe sweating and bronchorrhea after attempted suicide with *chumbinho*. He was treated with mechanical ventilation and continuous infusion of atropine. In a blood sample sent to the PCC, an absence of cholinesterase activity (0%) was detected on both the first and third days, suggesting the possibility of intoxication by an organophosphate insecticide. Clinical worsening was observed on the fourth day with fever and reversed cardiorespiratory arrest, and he died on the fifth day. Before the death was confirmed, he showed signs that could suggest uncontrolled cholinergic intoxication (miosis, sweating and bronchorrhea).

## DISCUSSION

The data presented show, as previously reported, that, although the frequency of toxic exposures in children under five is higher when compared to that of other age groups, the greater severity of intoxications is related to intentional exposures, which are more common in adolescents and adults.^7^

When we analyze the Brazilian reality about care provided by the PCCs, this does not allow a general comparison with the care reported by other countries. However, it is possible to compare the lethality rates/100 thousand inhabitants. Whereas in the United States, in 2016, the lethality rate/100 thousand inhabitants of SATE reported in the annual report of the American Association of Poison Control Centers (AAPCC) was 0.09/100 thousand inhabitants (<20 years old) and 0.1/100 thousand inhabitants (<5 years old).^7^ In the administrative region of Campinas, the rates detected in 2014/2015 were significantly higher, of 0.17/100 thousand inhabitants (<20 years old) and 0.24/100 thousand inhabitants (<5 years old). These data may suggest more precarious care for critically ill patients (children and adolescents) in our region of coverage.

Other variables, including iatrogenesis (see case 2), may have contributed to the lethal outcome. In Brazil, not all chemicals with the potential risk of causing serious local damage, such as corrosives and solvents, are packed in packaging with safety caps and, in many cases, are stored in non-original packaging, as described in the lethal case 2, apart from clandestine products.^6^ ^,^ ^10^ Packaging with child-proof safety lids has proven to be effective in the prevention of serious toxic exposures, especially in children <5 years old.^11^ ^,^ ^12^ Another variable that should be considered reflects the local epidemiology, such as the increasing incidence of severe and lethal cases of scorpionism in Brazil, mainly in the Southeastern and Northeastern regions, associated to the increase in the occurrence of poisonings caused by the yellow scorpion (*T. serrulatus*), a parthenogenetic species with a high capacity to adapt to the urban environment.^13^ In Brazil, in 2017, 124,662 cases of scorpionic accidents were reported in the Information System of Notifiable Diseases (SINAN), from the Ministry of Health, with 87 deaths related to the condition, 46% in children <10 years old.^14^

Other cases of severe poisoning were caused by rattlesnakes, possibly *Crotalus durissus terrificus*, the only subspecies present in our geographic region of care provided.^15^ All had a good evolution after treatment with anticrotalic antivenom and supportive measures, including adequate fluid replacement. Among snakebites caused by the four genera of venomous snakes in Brazil (*Bothrops*, *Crotalus*, *Lachesis* and *Micrurus*), envenomation caused by rattlesnakes (*Crotalus durissus* ssp.) is among the most serious, with 38 deaths among 4,160 cases reported in SINAN in 2015 and 2016 (0.9%).^14^ ^,^ ^15^

Another form of severe intoxication was related to the ingestion of pesticides used illegally as rodenticide, *chumbinho*. In Brazil, most cases of *chumbinho* poisoning are associated to cholinesterase inhibitors, such as carbamates (aldicarb and carbofuran).^16^ These data indicate that, in Brazil, even with the ban on commercial presentation of products containing aldicarb in 2012,^17^, there are still cases of serious poisoning by *chumbinho*, including other cholinesterase inhibitors, such as organophosphates. A challenge in the clinical management of these cases, in addition to life support measures, is the correct administration of atropine (muscarinic acetylcholine antagonist), which may require the use of high doses, for several days, until complete improvement of the cholinergic syndrome, especially when the active ingredients are organophosphates.^16^ Nonetheless, many doctors are resistant to the administration of high doses of atropine, a fact that can negatively interfere with the prognosis.^18^

Considering exposures to new psychoactive substances of illegal use, we highlight the case of severe intoxication by NBOMe (potent serotonergic agonist with stimulating and hallucinogenic effects), confirmed by GC-MS, in a 15-year-old girl admitted in another state and followed by our PCC. Most common adverse effects associated to exposure to NBOMe include agitation, tachycardia, hypertension, seizures and laboratory changes, including increased total CK (rhabdomyolysis). More serious cases can progress to kidney and respiratory failure,^19^ complications detected in the case we reported. As to psychoactive substances of consecrated recreational use, it is worth mentioning the fatal case of cocaine post-consumption resulting from hemorrhagic stroke, which is part of the set of vascular complications that can be detected after the consumption of cocaine hydrochloride or cocaine crack, such as acute myocardial infarction, ischemic or hemorrhagic stroke and aortic dissection.^20^ ^,^ ^21^

Another fatal case resulted from an accidental exposure to a pyrethroid insecticide in a commercial presentation containing organic solvents, confirmed by GC-MS (case 1). A similar case was described in a 53-year-old man with a lethal evolution after intentional ingestion of a product containing deltamethrin associated with a solvent containing naphtha, whose death was attributed, based on the clinical manifestations observed and the biological matrices analyzed *post-mortem*, to aromatic hydrocarbons.^22^

Medication poisoning was an important cause of SATE, mainly due to suicide attempts in adolescents and the association of drugs, such as anticonvulsants and antidepressants. Another serious drug intoxication involved an accidental ingestion of a topical decongestant derived from imidazoline (brimonidine; α2-adrenergic agonist) in a one-month-old newborn, due to administration error, which evolved with apnea and bradycardia; the boy was intubated before the consultation at the PCC. Similar cases evolve favorably within the first 24 hours without the need for invasive procedures, with improvement in episodes of apnea and bradycardia after intermittent tactile stimulation.^23^ ^,^ ^24^ In fatal case 4, clinical manifestations and severe hypoglycemia at admission suggest that an oral hypoglycemic intake may also have occurred.^25^

Impaction of button batteries in esophageal lumen can lead to high morbidity, including lethal outcomes.^26^ ^,^ ^27^ Generally, the most serious exposures occur after the ingestion of batteries ≥2 cm in diameter, such as 2032 lithium batteries, often without a report by caregivers, which can lead to delay in diagnosis and endoscopic removal, as well as in the prognosis of local injury.^26^ ^,^ ^27^ It should be noted that batteries lodged in the esophagus can cause serious injuries in the first two hours of contact with the mucosa. Local damage is basically caused by three mechanisms: direct pressure (pressure necrosis); leakage of the battery’s chemical content; and generation of electric current by contact of the battery poles with the esophageal mucosa (electrical burn).^26^ ^,^ ^27^

We use the PSS for severity classification. It was prepared by the European Association of Poison Centers and Clinical Toxicologists (EAPCCT), together with the International Program on Chemical Safety, of the World Health Organization. The collaborative work that validated the PSS involved 14 centers from various countries, and an agreement index obtained was above 80%.^8^ The PSS has no prognostic value and applies to acute toxic exposures, and must be established at the end of the case.^8^ Despite favorable and unfavorable criticisms,^28^ ^,^ ^29^ the PSS is simple and widely used.^29^

The present study has several limitations, which include the retrospective analysis of medical records and possible biases in the interpretation of the recovery of the data listed for analysis, data extracted by the authors in a non-blind manner. In addition, most of the PCC service information was obtained from telephone consultations, which may have been incomplete. Moreover, in the review of electronic medical records, there were flaws in the interpretation of the PSS classification to the outcomes, leading to the exclusion of 11 cases for final analysis.

Despite these limitations, the authors report good quality in filling out all electronic medical records, as well as the practical interface for retrieving data from the Datatox system. Finally, the results presented confirm the importance of the work of a PCC of regional reference in the urgency and emergency care network,^30^ which can serve as a basis for preventing and training health teams to manage these cases, aiming to improve care in handling these injuries.

We conclude that venomous animals’ sting/bites, poisoning by medicines and chemicals for household/industrial use were the main causes of SATE, significantly more frequent in adolescents, mainly due to suicide attempts.
